# LRRK2 Modulates the Exocyst Complex Assembly by Interacting with Sec8

**DOI:** 10.3390/cells10020203

**Published:** 2021-01-20

**Authors:** Milena Fais, Giovanna Sanna, Manuela Galioto, Thi Thanh Duyen Nguyen, Mai Uyên Thi Trần, Paola Sini, Franco Carta, Franco Turrini, Yulan Xiong, Ted M. Dawson, Valina L. Dawson, Claudia Crosio, Ciro Iaccarino

**Affiliations:** 1 Department of Biomedical Sciences, University of Sassari, 07100 Sassari, Italy; faismilena@gmail.com (M.F.); sanna.posta@gmail.com (G.S.); galioto@uniss.it (M.G.); nguyenthanhduyen88@gmail.com (T.T.D.N.); uyenthimai@gmail.com (M.U.T.T.); sinipaoladrop@gmail.com (P.S.); ccrosio@uniss.it (C.C.); 2 Nurex Srl, 07100 Sassari, Italy; franco.carta@nurex.it (F.C.); francesco.turrini@unito.it (F.T.); 3 Department of Oncology, University of Turin, 10126 Turin, Italy; 4 Neuroregeneration and Stem Cell Programs, Institute for Cell Engineering, Johns Hopkins University School of Medicine, Baltimore, MD 21205, USA; yxiong@uchc.edu (Y.X.); tdawson@jhmi.edu (T.M.D.); vdawson@jhmi.edu (V.L.D.); 5 Department of Neurology, Johns Hopkins University School of Medicine, Baltimore, MD 21205, USA; 6 Department of Pharmacology and Molecular Sciences, Johns Hopkins University School of Medicine, Baltimore, MD 21205, USA; 7 Solomon H. Snyder Department of Neuroscience, Johns Hopkins University School of Medicine, Baltimore, MD 21205, USA; 8 Department of Physiology, Johns Hopkins University School of Medicine, Baltimore, MD 21205, USA

**Keywords:** LRRK2, Sec8, exocyst complex, Parkinson’s disease

## Abstract

Mutations in LRRK2 play a critical role in both familial and sporadic Parkinson’s disease (PD). Up to date, the role of LRRK2 in PD onset and progression remains largely unknown. However, experimental evidence highlights a critical role of LRRK2 in the control of vesicle trafficking, likely by Rab phosphorylation, that in turn may regulate different aspects of neuronal physiology. Here we show that LRRK2 interacts with Sec8, one of eight subunits of the exocyst complex. The exocyst complex is an evolutionarily conserved multisubunit protein complex mainly involved in tethering secretory vesicles to the plasma membrane and implicated in the regulation of multiple biological processes modulated by vesicle trafficking. Interestingly, Rabs and exocyst complex belong to the same protein network. Our experimental evidence indicates that LRRK2 kinase activity or the presence of the LRRK2 kinase domain regulate the assembly of exocyst subunits and that the over-expression of Sec8 significantly rescues the LRRK2 G2019S mutant pathological effect. Our findings strongly suggest an interesting molecular mechanism by which LRRK2 could modulate vesicle trafficking and may have important implications to decode the complex role that LRRK2 plays in neuronal physiology.

## 1. Introduction

Mutations in the leucine-rich repeat kinase 2 gene (LRRK2, PARK8) are the most frequent genetic causes of Parkinson’s disease, reaching up to 40% in some ethnic groups such as Ashkenazi Jewish and North African Arab Berbers [1]. These LRRK2 pathological mutations are autosomal dominant and PD patients carrying the LRRK2 mutations are clinically and neuropathologically indistinguishable from idiopathic patients [2,3]. LRRK2 belongs to the Roco superfamily of proteins, which constitutes a novel multi-domain family of Ras-like G-proteins. LRRK2 is a large multidomain protein consisting of armadillo repeats (ARM), ankyrin repeats (ANK), leucine-rich repeats (LRR), Ras of complex (Roc), *C*-terminal of Roc (COR), kinase, and WD40 domains [4]. LRRK2 mutations are high frequent around the central catalytic core of the protein, two mutations are found in the Roc domain, one in the COR domain and two in the kinase domain. In addition, two variants that act as risk factors for sporadic PD have been identified, one in the COR domain and one in the WD40 repeats [4].

Despite the apparent clinical association between LRRK2 mutations and PD, it remains enigmatic how LRRK2 pathological mutations may contribute to disease onset and progression. Different experimental evidence suggests that LRRK2 has a functional role in the vesicle trafficking control, and alteration in synaptic vesicle trafficking seems a common pathological mechanism in PD [5,6]. In fact, many LRRK2 protein interactors belong to protein families involved in the regulation of vesicle trafficking (among them Rab5 [7], Rab7 [8], Rab7L [9,10], Sec16A [11], a subset of Rabs [12], endoA [13]) or of cytoskeleton dynamics that in turn may modulate vesicle trafficking [14,15,16,17]. Since one of the first deep analyses by Piccoli et al. in 2011 where LRRK2 controls synaptic vesicle storage and mobilization within the recycling pool [18], hundreds of different publications have underlined the role of LRRK2 vesicle trafficking. Mutant LRRK2s alter endocytosis by the phosphorylation of DNAJC6 (auxilin) [19], synaptojanin1 [19], endoA [13], or Rab5b [7] and modulate vesicle dynamics via aberrant phosphorylation of NSF [20] or different Rab family proteins. Recently, a significant reduction in synaptic vesicle number and a greater abundance of clathrin-coated vesicles were observed in a mouse line expressing tetracycline-inducible LRRK2 G2019S in catecholaminergic neurons, [21]. We have previously demonstrated that LRRK2 modulates dopamine receptor trafficking [22,23]. LRRK2 mutations dramatically modify the excitatory synaptic activity with a fourfold increase in sEPSC frequency in the dorsal striatal spiny projection neurons altering the shape of postsynaptic structures into striatum [24]. Moreover, in G2019S LRRK2 KI mice, elevated glutamate, and dopamine transmission and aberrant D2-receptor responses are detected independent of any change in the number of synapses or spine-like structures [25]. Glutamate terminals within the striatum are subject to active neuromodulation by other neurotransmitters released in the local area, including dopamine. Moreover, growing evidence underlines overlapping genes involved in both SV dynamics and autophagy, suggesting that vesicle trafficking has also an important function in the regulation of autophagic processes (for review see [26]).

In this context, the exocyst complex is an evolutionarily conserved multisubunit protein complex mainly implicated in tethering secretory vesicles to the plasma membrane. The exocyst complex is highly conserved in eukaryotic systems and is composed of eight single-copy subunits: Sec3, Sec5, Sec6, Sec8, Sec10, Sec15, Exo70, and Exo84 [27]. Genetic and biochemical studies in yeast indicate that the exocyst functions upstream of SNAREs. Different members of the exocyst complex interact with SNARE members or SNARE interacting proteins. In particular, yeast Sec6 can interact with the t-SNARE protein Sec9 (the SNAP-25 homolog) and with the Sec1/Munc18 family protein Sec1 [28]. These interactions are thought to precede vesicle priming, a process mediated by SNAREs (t-SNAREs on plasmatic membrane and v-SNARE on vesicle membrane) to dock the vesicles to the receiving membrane and finally to induce lipid fusion [29]. Importantly, mutations in the exocyst component Sec5 mainly alter the cell growth and membrane protein insertion without significant alteration in neurotransmitter release [30]. Axon and dendrite outgrowth relies on continuous membrane expansion and cytoskeletal remodeling and, in fact, neurite outgrowth is impaired in the absence of functional exocyst subunits in various biological systems such as primary neurons, PC12 cells, or multicellular model organisms [31,32,33] highlighting the prominent role played by the exocyst in the determination of neuronal cell polarity. Interestingly exocyst and Rabs (including Rab8 and Rab10, two LRRK2 phosphorylation targets) are part of the same protein complex that couples the generation of secretory vesicles at donor compartments to their docking and fusion [34].

The gene encoding for Sec8 is a 110-kDa multidomain protein containing 974 amino acids. Sec8 is expressed throughout the brain, and there is no significant regional variation in the different brain areas. Knockout of the Sec8 gene in mice is early embryonic lethal; mutant embryos initiate gastrulation but are unable to progress beyond the primitive streak stage [35]. Interestingly, Sec8 binds to postsynaptic density protein 95 (PSD95) by the *C*-terminal region, which contains a PDZ binding domain. PSD95 is a synaptic scaffolding protein that plays a pivotal role in synaptic plasticity [36]. Moreover, Sec8 is essential for appropriate targeting to the cell membrane of the α-amino-3-hydroxy-5-methyl-4-isoxazole propionic acid receptor (AMPAR) and the *N*-methyl-d-aspartate receptor (NMDAR) [37]. This last effect is mediated by the interaction of the Sec8 PDZ binding domain with the Synapse-associated protein (SAP) 102 [37]. Finally, exo70 and Sec8 subunits of the exocyst complex directly associate with the intracellular domain of NCAM140 [38]. NCAM promotes FGF receptor-mediated phosphorylation of two tyrosine residues in the Sec8 protein and is required for efficient recruitment of the exocyst complex to growth cones [38]. Sec8 is associated with various biological processes, such as cell migration, invadopodia formation, cytokinesis, glucose uptake, and neural development. It plays a crucial role in targeting intracellular vesicles to their sites of fusion with plasma membrane both during neurite outgrowth and synaptogenesis as well as in mature synapses [39]. In neurons, this process is crucial since both the regulated protein localization on the plasma membrane of axon or dendrites (e.g., neurotransmitter or neurotrophic factor receptors) and the correct release of proteins (e.g., neurotransmitters or neurotrophic factors) mediate neuronal communication and underlie virtually all functions of the nervous system.

In the present study, we show that LRRK2 associates Sec8 and modulates the exocyst complex assembly. Importantly, the LRRK2 effect is mediated by its kinase domain since it is significantly impaired by LRRK2 kinase inhibitor treatment or by the absence of the LRRK2 kinase domain itself. Finally, the over-expression of Sec8 can significantly rescue the LRRK2 G2019S pathological mutant phenotype in neuronal cellular systems.

## 2. Materials and Methods

### 2.1. Reagents and Solutions

Tween^®^ 20 (Polyethylene glycol sorbitan monolaurate), protease inhibitor cocktails, IGEPAL^®^ CA-630 (Octylphenoxy poly(ethyleneoxy)ethanol), LRRK2 inhibitors: CZC-25146 from Merck (Darmstadt, Germany) The phosphate-buffered saline (PBS) solution was made using NaCl (137 mM), KCl (2.7 mM), Na2HPO4 (8.1 mM), KH2PO4 (1.47 mM) from Merck (Darmstadt, Germany) and then adjusted to pH 7.4. Dulbecco’s modified Eagle’s medium (DMEM)–F12, Fetal Bovine Serum (FBS), Streptomycin/Penicillin, Geneticin-G418 were purchased from ThermoFisher Scientific (Waltham, MA, USA).

### 2.2. Plasmid Constructions

Plasmids coding WT or mutants LRRK2 were previously described in [22]. LRR, Kinase, and WD40 domains were cloned in frame with GST into pGEX plasmid after PCR amplification using the following forward and reverse oligos: LRR (aggatccgtgttcatttggagcatc and aggatccccaaaggaagatcccata), Kinase (aggatcctggctgacctgcctagaa and aggatcctgcgtctcgtcagacaga), WD40 (aggatccacagcaggaatgcaagca and aggatccgcacttcatgtggaagat). After PCR amplification both pGEX plasmid and PCR products were cut by BamHI and ligated.

Rat Sec8 cDNA was a generous gift from Dr. Lienhard [40]. Human Sec8 was PCR amplified and cloned in frame with Flag-tag in the *N*-terminal position into pcDNA3 plasmid. Sec8 deletion mutants were generated by PCR following the QuikChange II Site-Directed Mutagenesis Kit using the following forward and reverse oligonucleotides (Sec8-Δ1: ggcagaagcagcttacatcaaatcgact and acatcaaatcgactcaagatgttctaca; Sec8-Δ2: acatcaaatcgactcaagatgttctaca and tgtagaacatcttgagtcgatttgatgt; Sec8-Δ3: ccaagatgttcatcctctcctacagag and ctctgtaggagaggtagaacatcttgg; Sec8-Δ4: ctctcctacagagctgcttgcttgtctt and aagacaagcaagcagctctgtaggagag; Sec8-Δ5: cttgcttgtcttaaagaagataactac and gtagttatcttctttaagacaagaag.

### 2.3. Cell Lines

Human neuroblastoma SH-SY5Y cells (ATCC number CRL-2266) were grown in DMEM-F12 (ThermoFisher Scientific, Waltham, MA, USA), 10% fetal calf serum (FCS) (ThermoFisher Scientific, Waltham, MA, USA) at 37 °C. The PC12-TET-ON (Takara Bio Inc, Shiga, Japan) and PC12-TET-ON-G2019S cell lines were cultivated in DMEM-F12 supplemented with 10% Tetracycline-free FCS (Lonza, Milano, Italy) at 37 °C. HEK 293T (ATCC number CRL-3216) were grown in DMEM-F12 (Thermo Fisher Scientific), 10% fetal calf serum (FCS, Thermo Fisher Scientific) at 37 °C.

### 2.4. GST Pull-Down Assay

GST-pull down was previously described [41]. Briefly, GST-LRR, Kinase, or WD40 fusion domains were expressed in *E. coli* BL21. After induction with 0.5 mM IPTG for 3 h at 30 °C, bacteria were collected, washed twice in PBS1X and lysed by sonication (5″ at 100 W for 3 times) in lysis buffer (50 mM Tris HCl pH 8.0, 1 mM EDTA, 150 mM NaCl, 1% Triton X100, 1 mM PMSF, protease inhibitor cocktail 1X, 200 μg/mL lysozyme). GST-fused proteins were purified on glutathione–Sepharose 4 fast flow resin (GE Healthcare, Buckinghamshire, UK). Aliquots (500 ng) of the three GST-LRRK2 domains or GST alone were used for GST-pull down experiments. 5 mg of mouse brain protein extracts, obtained by homogenization in lysis buffer (50 mM Tris–HCl pH 8.0, 150 mM NaCl, 1% NP-40, 1 mM PMSF, protease inhibitor cocktail 1X) were incubated with GST-LRRK2 domains at 4° O/N. After five washes in lysis buffer, the beads were boiled in Laemmli Buffer 1X and loaded on SDS/PAGE acrylamide gel. Finally, the gels were stained with Mass Compatible Super Blue Stain Kit (Nurex Srl, Sassari, Italy).

### 2.5. In-Gel Digestion and Matrix-Assisted Laser Desorption/Ionization (MALDI) Mass Spectrometry (MS) Analysis

The mass spectrometry analysis was performed as described [41]. Briefly, the protein of interest was excised with a sterile scalpel and destained with 100 μL of 5 mM NH_4_HCO_3_/50% acetonitrile. Gel pieces were treated with 10 μL of 5 mM NH_4_HCO_3_ containing 10 ng/μL trypsin for 40 min on ice. Subsequently, excess digestion buffer was removed and substituted with an equal volume of 5 mM NH_4_HCO_3_. Tryptic digestion was conducted overnight at 37 °C. The MS spectra acquired were submitted to MASCOT (Matrix Science, London, UK).

### 2.6. Subcellular Fractionation of HEK293 Cells

Subcellular fractionation was performed as previously described in [42]. Briefly, the cells were homogenized in ice-cold homogenization-buffer (320 mM sucrose, 4 mM HEPES, pH 7.4, protease inhibitor cocktail (Sigma). An aliquot of cell lysate was used as a total fraction in the western blot. The homogenates were centrifuged at 1000× *g* for 10 min to produce a pellet containing nuclei and large debris fraction that was discarded. The supernatant was further fractionated into a pellet (containing the membrane fraction) and supernatant by centrifugation at 10,000× *g* for 20 min. The membrane fraction was further washed by ice-cold homogenization-buffer to eliminate any cytoplasmic protein. The supernatant was ultra-centrifuged at 60,000× *g* for 60 min to obtain the pellet (containing the vesicle fraction) and the supernatant containing the cytoplasmic fraction. The vesicle fraction was further washed by ice-cold homogenization-buffer to obtain pure vesicles. Protein content was determined using the Bradford protein assay. An equal amount of protein extracts was loaded into the SDS-PAGE.

### 2.7. Immunoprecipitation

HEK293 cells were transfected with the indicated plasmids in 6 cm cell culture plates. The LRRK2 inhibitor CZC25146 (Merck, Darmstadt, Germany) was added 3 h before cell lysis at a final concentration of 1 μM. After 48 h the cells were washed twice in PBS 1X and lysed by 1 mL of NP40 lysis buffer (150 mM NaCl, 1% NP40, 20 mM Tris-HCl pH 7.5, protease inhibitor cocktail). Cellular debris were removed by centrifugation at 13,000× *g* and cell lysates were pre-cleared by incubation with protein A-agarose beads for 1 h at 4 °C. Then the samples were incubated by the indicated antibodies (anti-Flag, F3165, 1:2000 Merck, Darmstadt, Germany) or anti-Myc, M4439, 1:2000, Merck, Darmstadt, Germany)) overnight at 4 °C. After incubation with protein A-agarose for 1 h at 4 °C, the beads were washed 4 times by lysis buffer. Samples were then resolved by SDS-PAGE.

### 2.8. Western Blot Analysis

Western blot analysis was performed as previously described [22]. Briefly, protein extracts were prepared by direct lysis in Laemmli buffer or NP40 1% buffer when protein content was determined using the Bradford protein assay. Equal amounts of protein extracts were resolved by standard sodium dodecyl sulfate-polyacrylamide gel electrophoresis and subsequently electroblotted into nitrocellulose membrane (Thermo Fisher Scientific). Membranes were incubated with 3% low-fat milk in 1X PBS-Tween 0.05% solution with the indicated antibody: anti-LRRK2 (1:5000 MJFF2 c41-2 ABCAM, Cambridge, UK), anti-Myc (1:5000 M4439 Sigma-Aldrich), anti-Flag (1:2500 F3165 Sigma-Aldrich), anti-beta-actin (A5441 1:5000 Sigma-Aldrich), anti-Sec8 (1:1000 610659 BD Biosciences, San Jose, CA, USA), anti-Exo70 (1:1000 HPA022840 Sigma-Aldrich), anti-Sec6 (1:1000 SAB2100729 Sigma-Aldrich) for 16 h at 4 °C. Goat anti-mouse immunoglobulin G (IgG) peroxidase-conjugated antibody (1:2500 Merck, Darmstadt, Germany) or goat anti-rabbit IgG peroxidase-conjugated antibody (1:5000 Millipore Corporation) were used to identify immunocomplexes by enhanced chemiluminescence (ECL start, Euroclone SpA, Milano, Italy).

### 2.9. Immunofluorescence

The cells were grown on a cover-glass, washed twice with PBS 1X, and then fixed with 4% paraformaldehyde/PBS for 10 min. Cells were permeabilized with 0.1% Triton X-100 diluted in PBS. Non-specific binding was blocked with 5% bovine serum albumin, 0.05% Tween-20 diluted in PBS for 1 h at room temperature. Cells were incubated with primary antibodies: anti-LRRK2 (1:500 Mjff2 c41-2 Epitomics), anti-Flag (F3165 1:2000 Sigma-Aldrich), anti-Myc (M4439 1:2000, Sigma-Aldrich) diluted in blocking solution, overnight at 4 °C. Cells were then washed with PBS, 0.05% Tween-20 diluted in PBS and incubated with secondary antibodies: Goat anti-Mouse IgG Secondary Antibody Alexa Fluor^®^ 488 (ThermoFisher Scientific, Waltham, MA, USA) and Goat anti-Mouse IgG Secondary Antibody Alexa Fluor^®^ 647 (Life Technologies) diluted 1:1000 in blocking solution for 1 h at room temperature. Before analysis, cells were mounted using Mowiol mounting medium, and fluorescence was revealed with a Leica TCS SP5 confocal microscope with LAS lite 170 image software.

### 2.10. Disuccinimidyl Suberate (DSS) Cross-Linking

The experiment was performed following the manufactory’s instructions (21555 ThermoFisher Scientific, Waltham, MA, USA). Briefly, ~2.5 × 10^6^ cells in 6 cm plates were washed by PBS1X, collected in PBS1X using a scraper, and resuspended in 100 microliters of PBS1X. DSS solution to a final concentration of 2 mM was added and incubated for 30 min at room temperature. Finally, the cells were lysed by 1 mL of NP40 lysis buffer (150 mM NaCl, 1% NP40, 20 mM Tris-HCl pH 7.5, protease inhibitor cocktail (Roche, Basel, Switzerland)), and protein extracts were used for immunoprecipitation experiments as previously described.

### 2.11. Statistical Analysis

The results are presented as means ± SEM. of independent experiments as indicated. Statistical evaluation was conducted by One-way ANOVA and Bonferroni’s multiple comparison post-test. Values significantly different from the relative control are indicated with one, two, or three asterisks when *p* < 0.05, *p* < 0.005, and *p* < 0.001, respectively.

## 3. Results

### 3.1. LRRK2 Interacts with Sec8

**Figure 1:**
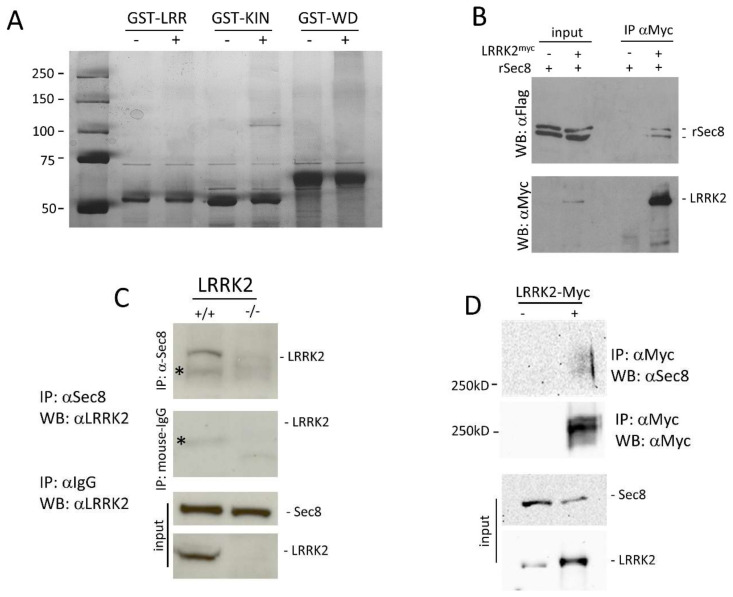
Analysis of LRRK2 and Sec8 physical interaction and colocalization. (**A**) 5 mg of mouse brain protein extracts were used for a GST pull-down experiment using three different LRRK2 domains: LRR, Kinase, and WD40. The protein complexes were separated by reducing SDS-PAGE and subjected to sensitive colloidal Coomassie Brilliant Blue staining. (**B**) HEK293 cells were transfected by ratSec8-Flag or co-transfected by Flag-ratSec8/Myc-LRRK2 WT for 48 h. The cells were lysed and the protein extracts were subjected to a co-immunoprecipitation experiment using an anti-Myc antibody. Total and immunoprecipitated proteins were visualized by western blot using an anti-Flag antibody to visualize ratSec8. Then the membrane was incubated by an anti-Myc to evaluate the immunoprecipitation efficacy. (**C**) Mouse brain protein extracts from WT or LRRK2 knock-out mice were subjected to a co-immunoprecipitation experiment using an anti-Sec8 or anti-IgG antibody and the immunocomplex were revealed by an anti-LRRK2 antibody. Anti-Sec8 and anti-LRRK2 were also used on the input fraction as the control for equal protein amount. * indicates a nonspecific band. (**D**) HEK293 cells were transfected or not by Myc-LRRK2 WT for 48 h. The cells were treated by DSS and protein extracts were subjected to a co-immunoprecipitation experiment using an anti-Myc antibody. Immunoprecipitated proteins were visualized by western blot using an anti-sec8 antibody to visualise endogenous Sec8. Then the membrane was incubated by an anti-Myc to evaluate the immunoprecipitation efficacy. The protein levels of endogenous Sec8 and endogenous or transfected LRRK2-Myc were visualized by anti-Sec8 and anti LRRK2 respectively.

To identify LRRK2 protein interactors we decided to realize a GST-pull down approach. We subcloned three different LRRK2 domains (LRR, kinase, or WD40 domain) in frame with GST to generate GST-LRR, GST-Kinase, or GST-WD40 constructs. All recombinant proteins were expressed in the BL21 strain of E. coli and purified by affinity chromatography. The purified proteins (GST-LRR, GST-WD40, or GST-Kinase) were used in GST pull-down experiments with 5 mg of mouse brain protein extracts. The mouse brain proteins interacting with the different GST-fused LRRK2 domains were separated by SDS-PAGE and identified by Mass Spectrometry. Interestingly we found that the GST-Kinase domain (but not the GST-LRR or GST-WD40) associates specifically with a protein of roughly 110 kD (Figure 1A). Mass spectrometry analysis permitted to identify this protein as Sec8, a member of the exocyst complex.

The interaction between LRRK2 and Sec8 was validated through two different co−immunoprecipitation experiments. HEK293 cells were transfected by a plasmid construct coding Flag-ratSec8 or co-transfected by LRRK2-Myc and Flag-ratSec8. Using protein extracts prepared 48 h after transfection, we performed a co-immunoprecipitation experiment using an anti-Myc antibody for immunoprecipitation and anti-Flag antibody for the subsequent western blot analysis of the complexes. As illustrated in Figure 1B the immunoprecipitation by anti-Myc pulls down specifically Flag-ratSec8 (Figure 1B upper). Note that ratSec8 appears as a doublet band in a typical SDS-PAGE analysis [40]. The filter was then probed by an anti-Myc antibody to control the LRRK2 expression level and immunoprecipitation efficacy (Figure 1B lower).

To ensure that our results in HEK293 cells were physiologically relevant at protein endogenous levels, we performed a second co-immunoprecipitation experiment using striatal brain tissues using commercially available antibodies against LRRK2 or Sec8. The Sec8 immunoprecipitation pulls down LRRK2 in WT (+/+) but not in the LRRK2 knock-out (−/−) mouse brain (Figure 1C). To further confirm the LRRK2/Sec8 interaction we performed a cross-linking experiment using the membrane-permeable compound disuccinimidyl suberate (DSS). HEK293 cells were transfected by LRRK2-Myc for 48 h. Before lysis, the cells were treated with DSS, and then the protein extracts were used for a co-IP experiment using an anti-Myc antibody. As shown in Figure 1D endogenous Sec8 appears as a protein smear of molecular weight higher than 250 kD. All these findings clearly indicate the spatial proximity of LRRK2 and Sec8 strongly suggesting that they are part of a common protein complex.

**Figure 2:**
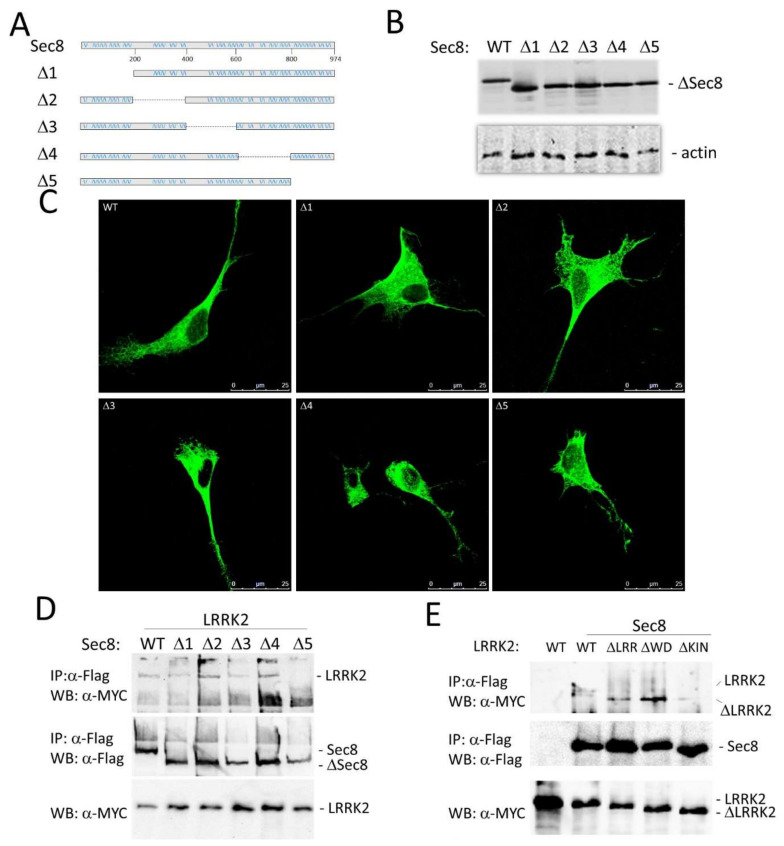
Identification of Sec8 protein region interacting with LRRK2. (**A**) Schematic representation of the Sec8 five deletion mutants generated. (**B**) HEK 293 cells were transfected by the different Sec8 deletion mutants. 48 h after transfection, the protein extracts were analyzed by western blot using an antibody against Flag epitope to detect Sec8. (**C**) HEK293 cells were transfected with the five different Flag-Sec8 deletion mutants for 48 h. After fixation, the cells were incubated with anti-Flag primary antibody and with Alexa488-conjugated secondary antibody (green). (**D**) HEK293 cells were co-transfected by Flag-Sec8 WT or the Sec8 deletion mutants and Myc-LRRK2 WT for 48 h. The cells were lysed and the protein extracts were subjected to a co-immunoprecipitation experiment using an anti-Flag antibody. Total and immunoprecipitated proteins were visualized by western blot using an anti-Myc antibody to visualize LRRK2. Then the membrane was incubated by an anti-Flag to evaluate the immunoprecipitation efficacy. (**E**) HEK293 cells were transfected with Myc-LRRK2 WT or co-transfected with Myc-LRRK2 WT or LRR (ΔLRR) or Kinase (ΔKin) or WD40 (ΔWD40) deletion mutants and Flag-Sec8 for 48 h. The cells were lysed and the protein extracts were subjected to a co-immunoprecipitation experiment using an anti-Flag antibody. The co-immunoprecipitated proteins were visualized by western blot using an anti-Myc antibody to visualize LRRK2. Then the membrane was incubated by an anti-Flag to evaluate the immunoprecipitation efficacy. The input fraction was incubated by anti-Myc as the control for equal transfection efficacy of the different samples.

Human Sec8 is a protein of roughly 110 kD (974aa) with not well-defined functional domains. To identify the Sec8 protein region interacting with LRKK2 we decided to create five different Flag-hSec8 deletion mutants. Each mutant is devoid of roughly 200 amino acids (Figure 2A). All Sec8 deletion mutants are stable when expressed in HEK293 cells (Figure 2B) with no gross alteration in the cytoplasmic localization (Figure 2C).

Then we performed a co-immunoprecipitation experiment co-transfecting HEK293 cells by Myc-LRRK2 with one of the different Sec8 deletion mutants. As shown in Figure 2D the lack of the *C*-terminal part of Sec8 protein (Δ5) leads to the loss of LRRK2 protein interaction. We repeated a similar experiment using LRRK2 deletion mutants [43]. The absence of the kinase domain of LRRK2 strongly reduces the Sec8 binding (Figure 2E and Figure S1A) although this interaction still slightly occurs suggesting a more intricate LRRK2/Sec8 interaction involving other LRRK2 protein domains.

Once validated the LRRK2/Sec8 protein interaction, we have analyzed the potential role of LRRK2 in modulating Sec8 phosphorylation. Sec8 has been identified as an interactor using the kinase domain of LRRK2, therefore it was reasonable to suppose that Sec8 could be a direct substrate of LRRK2 kinase activity. Although we used different experimental approaches including a phosphorylation site identification by a Mass Spectrometry covering roughly 75% of the Sec8 protein, we were not able to highlight any significant change in Sec8 phosphorylation level due to the presence of LRRK2 WT or G2019S pathological mutant (data not shown).

### 3.2. LRRK2 Modulates the Exocyst Complex Assembly

**Figure 3:**
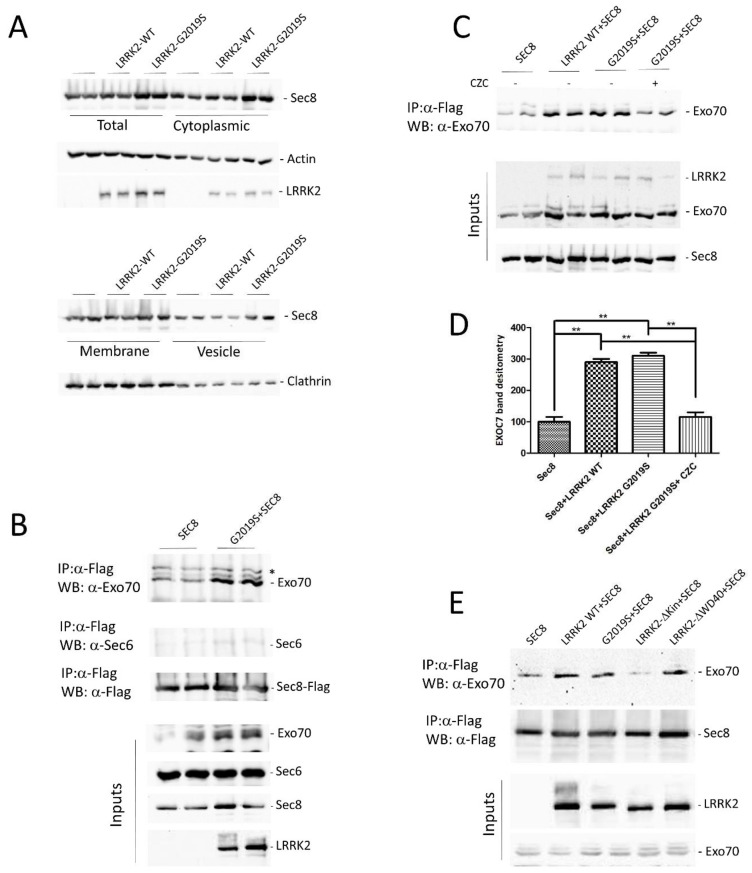
Evaluation of LRRK2 effect on Sec8 sub-cellular localization and exocyst assembly. (**A**) HEK 293 cells were transfected or co-transfected by the indicated plasmids. 48 h after transfection, different subcellular fractions (total, cytoplasm, membranes, and vesicles) were separated by differential centrifugation and analyzed by western blot using an antibody against Flag epitope to detect Sec8. Anti-β-actin and clathrin antibodies were used as loading control while anti-Myc was used to detect transfected LRRK2. (**B**) HEK293 cells were transfected or co-transfected for 48 h with Flag-Sec8 WT or Myc-LRRK2 G2019S as indicated. The cells were lysed and the protein extracts were subjected to a co-immunoprecipitation experiment using an anti-Flag antibody to isolate Sec8. The co-immunoprecipitated proteins were visualized by western blot using anti-Exo70 or Sec6 antibodies. Then the membrane was incubated by an anti-Flag to evaluate the Sec8 immunoprecipitation efficacy. The input fraction was incubated by anti-Exo70, Sec6 as a loading control, and anti-Flag or Myc as control of transfection efficacy. * indicates a nonspecific band (**C**) Co-immunoprecipitation experiment as in (**A**). The LRRK2 kinase inhibitor (CZC 25146) was added 3 h before cell lysis. (**D**) Relative band densitometry for Exo70 of data obtained in (**C**) normalized to cells transfected by Sec8 alone. The data represent the mean ± SEM of three independent experiments. ** *p* < 0.01. One-way ANOVA and Bonferroni post-test were used. (**E**) Co-immunoprecipitation experiment as in (**A**) in the presence of two different LRRK2 deletion mutants: Kinase (ΔKin) or WD40 (ΔWD40) domains.

To explore the consequence of LRRK2 expression on Sec8 function, we have evaluated the LRRK2 effect on Sec8 sub-cellular localization, using a biochemical approach. Sec8 is a cytoplasmic protein distributed in the cytosol or associated with cytoplasmic membrane or vesicles [44]. To investigate any possible alteration in Sec8 subcellular distribution mediate by LRRK2, we transfected HEK293 cells with Sec8-Flag alone or in combination with LRRK2WT o G2019S mutant. 48 h after transfection the cells were lysed and four different cellular fractions were purified: total, cytosolic, membrane, or vesicle. As illustrated in Figure 3A neither the expression of LRRK2 WT nor G2019S could significantly alter the Sec8 subcellular distribution compared to the cells expressing Sec8 alone. Some apparent differences in Sec8 subcellular distribution are only due to differences in transfection efficacy as pointed out by the analysis of total fractions and by the statistical analysis (Figure S1B).

Then we have analyzed the association of exocyst complex in the presence of LRRK2. Sec8 associates in co-immunoprecipitation experiments with both Exo70 [45] and Sec6 [46], the other two members of the exocyst complex. In a preliminary experiment, we transfected HEK293 cells by Sec8-Flag alone or in the presence of LRRK2 G2019S. 48 h later, Sec8 was immunoprecipitated from protein extracts and the immuno-complexes were analyzed for the presence of Exo70 or Sec6. As shown in Figure 3B, in the presence of LRRK2 G2019S there is a significant increase in Sec8 association with Exo70 and to a less extent with Sec6. Then we repeated a similar experiment to evaluate any difference in Sec8-Exo70 association between LRRK2 WT or G2019S or in the presence of LRRK2 kinase inhibitor (CZC-25146). We confirmed that the presence of LRRK2 leads to an increase in Sec8/Exo70 interaction, with no significant differences between LRRK2 WT and G2019S mutant, and importantly this association is strongly reduced upon LRRK2 kinase inhibitor treatment (Figure 3C,D). Finally, to further explore the importance of the LRRK2 kinase domain in the exocyst complex assembly, we performed the same co-immunoprecipitation experiment using two different LRRK2 deletion mutants: delta-kinase and delta-WD40 domain. The lack of LRRK2 kinase domain significantly impairs the LRRK2 effect on SEC8/Exo70 association while no effect was detectable by the LRRK2 WD40 deletion mutant (Figure 3E and Figure S1C).

Taken together, our results underline a prominent role of LRRK2 in the modulation of exocyst complex association mainly mediated by LRRK2 kinase domain or activity.

### 3.3. Functional Role of LRRK2/Sec8 Interaction

**Figure 4:**
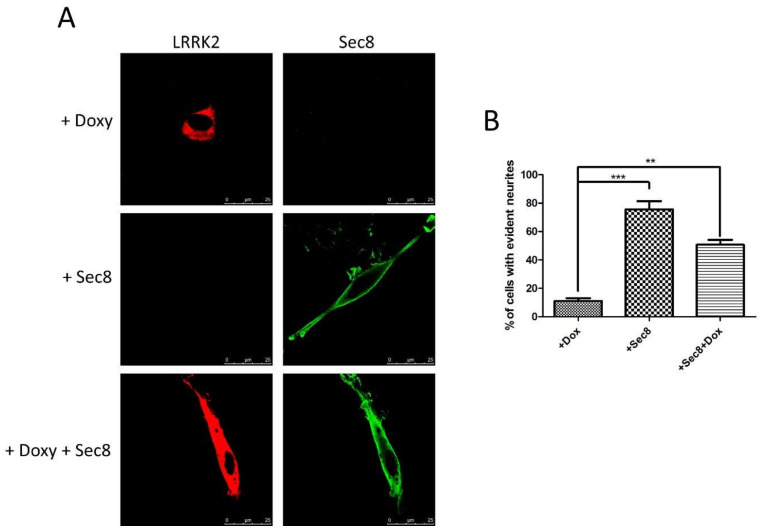
Analysis of Sec8 over-expression on neurite branching in PC12 or SH-SY5Y cells expressing LRRK2 G2019S. (**A**) PC12 cells stably expressing dox-inducible LRRK2 G2019S were transfected by Flag-Sec8 for 48 h and then treated for 6 days by NGF in the presence or absence of dox. (**B**) Quantification of data obtained in (**A**). The data represent the numbers of cells showing evident elongated cytoplasm in three independent experiments and are represented as mean ± SEM. At least 20 cells have been analyzed for each biological replicate. ** *p* < 0.01; *** *p* < 0.001. One-way ANOVA and Bonferroni post-test were used.

We reasoned that Sec8, when overexpressed, could strongly interact with LRRK2, likely blocking the LRRK2 physio-pathological effect on endogenous Sec8 or, in the alternative, Sec8 overexpression could overcome the LRRK2 effect on endogenous exocyst complex. The most common and easily detectable phenotype of LRRK2 pathological mutant expression is the impairment in neurite outgrowth in neuronal cells in different experimental models [17,47,48]. Therefore, we decided to analyze the effect of Sec8 over-expression on PC12 differentiation in the presence of LRRK2 G2019S pathological mutant. As previously published, PC12-ON cells stably expressing a doxycycline-inducible form of LRRK2 G2019S showed a significant impairment in neurite outgrowth due to Nerve Growth Factor (NGF) treatment [48]. After transfection with Sec8-Flag, we started the differentiation by NGF treatment for 6 days in the presence or absence of doxycycline to induce the LRRK2 G2019S expression. The differentiated cells were analyzed by immunofluorescence using antibodies against LRRK2 and Flag (for Sec8). The results confirmed that PC12-ON expressing LRRK2 G2019S (treated by doxycycline) show the typical round cellular phenotype of PC12 cells (Figure 4A upper panel), whereas those cells co-expressing Sec8 showed a significant change in cell body morphology with elongated/branched cytoplasm shape, a typical differentiation sign (Figure 4A lower panel). The PC12 cells expressing only Sec8 appeared well elongated (Figure 4A intermediate panel) without any significant difference with PC12-ON untransfected cells (data not shown). The data presented in Figure 4 were quantified in the bar graph of Figure 4B by analyzing a high number of cells.

The percentage of differentiated cells in the presence of LRRK2 G2019S (treated by doxycycline) was lower than 11% while the Sec8 over-expression led to a significant rescue (roughly 50% compared to 75% of cells expressing only Sec8). Similar results were obtained in another experimental system, the neuroblastoma SH-SY5Y cells differentiated by retinoic acid treatment (data not shown). The results strongly suggest that the over-expression of Sec8 may interfere with the LRRK2 G2019S pathological phenotype. To further highlight the change in PC12 cell morphology we acquired some images by phase-contrast microscopy (Figure S1D).

## 4. Discussion

Dominant variants of LRRK2 are considered the main genetic cause of hereditary parkinsonism, ranging from 1–6% of cases including many sporadic cases [4]. Different experimental results suggest an important role of LRRK2 in the control of vesicle trafficking, and alteration in synaptic vesicle trafficking seems a common pathogenetic mechanism in PD [6]. Interestingly, we describe a specific protein interaction between LRRK2 and Sec8, a member of the exocyst complex. LRRK2 and Sec8 protein complex was analyzed in transfected cell cultures and, most importantly, in the mouse brain at physiological protein levels (Figure 1C). Moreover, a cross-linking experiment further supports the LRRK2-Sec8 protein proximity (Figure 1D). The exocyst complex plays a crucial role in vesicle dynamics involved in a wide range of cellular functions. This octameric complex has been mainly implicated in the vesicular transport and recruitment to regions of rapid membrane growth, a process followed by SNARE-mediated vesicle fusion. In neurons, members of the exocyst are engaged in neurite outgrowth, and in cooperation with multidomain scaffolding proteins controls receptor transport to the synapse [39,45,49].

As mentioned in the introduction, exocyst and Rabs (including Rab8 and Rab10, two LRRK2 phosphorylation targets) are part of the same protein complex [34]. In their GTP-bound form, Rab proteins interact with downstream effectors, including exocyst members, controlling various steps of vesicle trafficking. For instance, Rab10 colocalizes and interacts with exocyst proteins, in particular Sec8, at the base of nascent cilia in renal epithelial cells [50]; Sec8 loss of function mutations cause proximal dendritic arborization defects and lead to the accumulation of intracellular Rab10 vesicles [51]; furthermore, Sec15, another member of the exocyst complex, interacts with Rab11 and affects Rab11 localization in vivo [52]. Therefore, we favor the hypothesis that the interaction between LRRK2 and Sec8 may explain different physio-pathological effects ascribed to LRRK2s including the cellular outcomes due to the LRRK2 modulation of Rab activity. We have identified the *C*-terminal region of Sec8 as the putative interaction region with LRRK2. Unfortunately, we could not use the Sec8 Δ5 deletion mutant for further experiments, since this mutant, although does not manifest gross alterations in the cellular localization (Figure 2C) shows a significant impairment in basal exocytosis (data not shown). This result is not surprising since Sec8 sequence analysis has shown that the Sec8 *C*-terminal region contains a type-1 PDZ-binding domain (TXV) that plays an important role in protein-protein interaction. For instance, this region regulates the interaction with two specific neuronal proteins: PSD-95 [36] and SAP102 [37], both concentrated at the post-synaptic density (PSD) of excitatory synapses and acting to assemble synaptic-signaling complexes.

Preliminary studies, mainly in yeast, had suggested that the exocyst complex can exist in two different sub-complexes: one contains Sec3, Sec5, Sec6, and Sec8 on vesicles, and the other contains Sec10, Sec15, Exo70, and Exo84 on the cell membrane [27]. The Sec3 and Exo70 localization at the plasma membrane seems dependent on the interaction with a phosphatidylinositol (4,5)-bisphosphate (PIP2) [53,54,55]. However, accumulating evidence questioned the existence of these two sub-complexes prior to the formation of the full complex since, for instance, the endogenous Sec3 protein shows a similar cellular distribution of the other exocyst subunits [56]. Furthermore, no sub-complexes could be isolated from S. cerevisiae, by more advanced biochemical studies [57]. In fact, Sec8 can interact with both Exo70 [45] and Sec6 [46] in co-immunoprecipitation experiments. Moreover, the Exo70 and Sec8 subunits are both associated with the intracellular domain of the neural cell adhesion molecule (NCAM) [38]. Based on these specific Sec8 interactions we decided to evaluate whether LRRK2 may affect the Sec8 association with Exo70 and Sec6. Interestingly, our experimental approach shows an increase in Sec8/Exo70 association in the presence of LRRK2. Furthermore, the LRRK2 effect is mediated by the LRRK2 kinase domain since it is significantly reduced either in the presence of LRRK2 kinase deletion mutant or by LRRK2 kinase inhibitor treatment. The effect of LRRK2 on Sec8/Sec 6 interaction in the co-immunoprecipitation experiment is less evident. The Sec6 co-IP is producing a very faint band although the antibody is well working in immunoblotting experiments. The result may be easily explained by the presence of Flag epitope or by the binding of the anti-Flag antibody both in the *N*-terminal position of Sec8. This Sec8 region is very relevant for Sec6 interaction since the deletion of the *N*-terminal domain of the Sec8 protein decreases its interaction with Sec6 while only slightly reduces its interaction with Exo70 [45]. The particular relevance of the LRRK2 kinase domain in modulating the Sec8/Exo70 interaction pushed us to evaluate any change in the phosphorylation level of Sec8 in the presence or absence of LRRK2 G2019S. Unfortunately, we were not able to identify any change in Sec8 phosphorylation either by in vitro kinase assay or by the analysis of Sec8 phospo-sites by Mass Spectrometry approach (data not shown).

Finally, we have explored the idea that Sec8, when over-expressed, could interact/sequester LRRK2 and eventually impair the LRRK2 G2019S pathological effect. Interestingly, Sec8 over-expression can significantly rescue the inhibition in differentiation due to LRRK2 G2019S expression (Figure 4). Future experiments are required to fully identify the molecular mechanism by which the LRRK2 kinase domain regulates exocyst complex assembly and function. However, our results agree with the suggested role of LRRK2 in the regulation of vesicle trafficking. Pharmaceutical compounds able to modulate vesicle trafficking may be a possible therapeutic option for PD treatment as recently suggested by the ability of Levetiracetam to rescue the LRRK2 G2019S pathological effect [48]. Levetiracetam acts by binding the SV2A protein located on synaptic vesicles and modulating the vesicle trafficking although by an unclear molecular mechanism. Recently, a new small molecule able to interfere with the exocyst complex has been identified: Endosidin2 (ES2) [58]. ES2 binds specifically to the Exo70 resulting in inhibition of exocytosis and endosomal recycling in both plant and human cells [58]. Could be worth designing specific experiments exploring the possible use of subliminal doses of ES2 compound in reducing the LRRK2 mediated toxicity in cellular and animal models.

## Data Availability

The data presented in this study are available on request from the corresponding author.

## Supplementary Materials

The following are available online at https://www.mdpi.com/2073-4409/10/2/203/s1.
