# Supplementary material for: LRRK2 Modulates the Exocyst Complex Assembly by Interacting with Sec8
Source: Cells. 2021 Jan 20;10(2):203. doi: 10.3390/cells10020203 (PMC7909581; doi:10.3390/cells10020203)
Supplement: Supplementary file 1 [file cells-10-00203-s001.pdf]

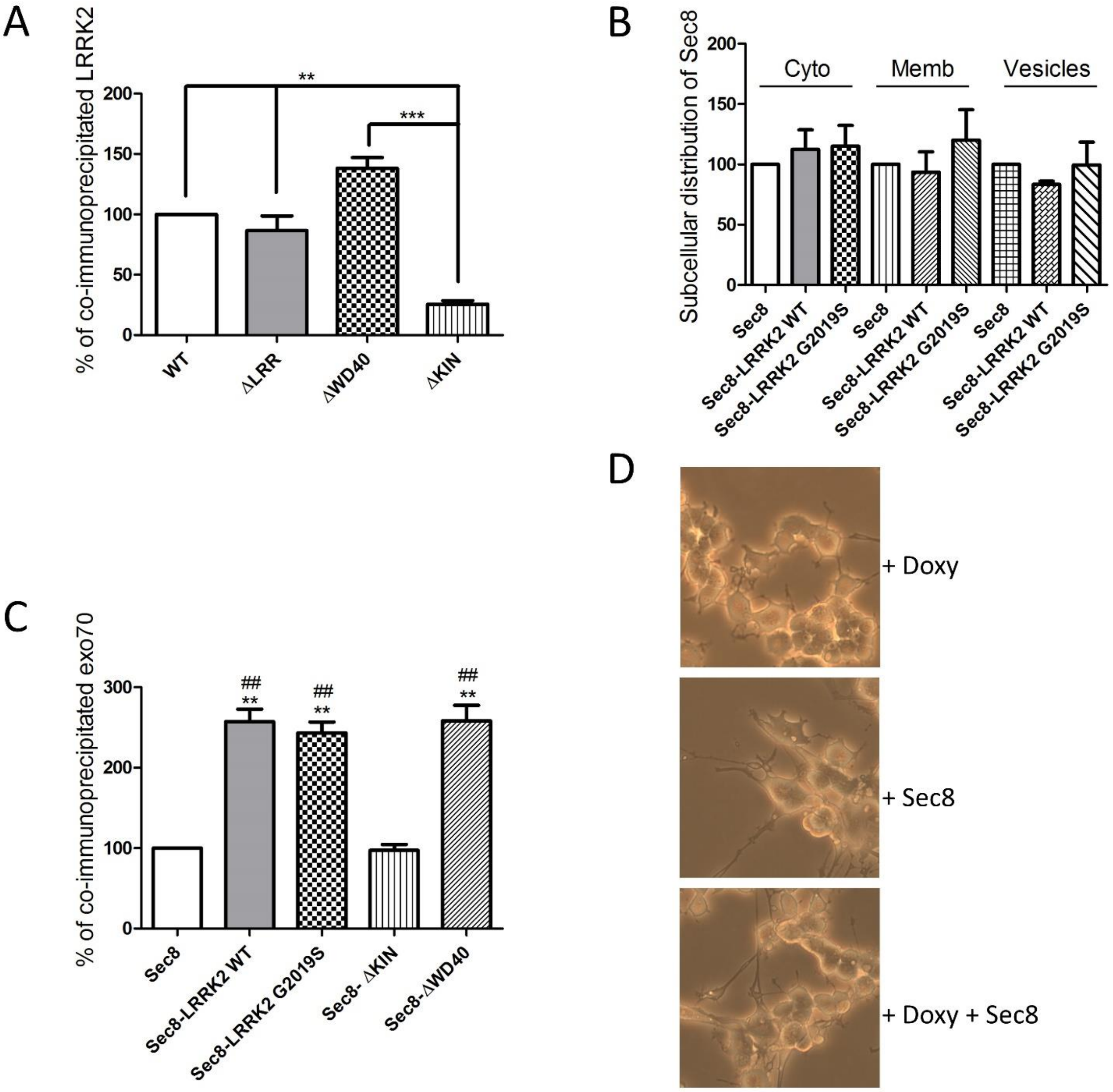

Supplementary Figure 1 (a) Relative band densitometry for the different LRRK2 isoforms of data obtained in (2E) indicating LRRK2 WT as 100%. The data represent the mean ± SEM of three independent experiments. **p<0,01, ***p<0,001. One-way ANOVA and Bonferroni post test were used. (**b**) Relative band densitometry for the Sec8 subcellular distribution of data obtained in (3A) indicating Sec8 alone as 100%. The data represent the mean ± SEM of three independent experiments. One-way ANOVA and Bonferroni post test were used. **(c)** Relative band densitometry for exo70 of data obtained in (3E) indicating the co-IP by sec8 alone as 100%. The data represent the mean ± SEM of three independent experiments. ** or ## p<0,01 vs Sec8 or Sec8-ΔKin respectively. One-way ANOVA and Bonferroni post test were used. **(d)** Representative picture of cell morphology change induced by NGF treatment in PC12 cells.
